# Leaf growth in early development is key to biomass heterosis in Arabidopsis

**DOI:** 10.1093/jxb/eraa006

**Published:** 2020-01-21

**Authors:** Pei-Chuan Liu, W James Peacock, Li Wang, Robert Furbank, Anthony Larkum, Elizabeth S Dennis

**Affiliations:** 1 Agriculture and Food, Commonwealth Scientific and Industry Research Organisation, Canberra, ACT, Australia; 2 Faculty of Science, University of Technology Sydney, Sydney, NSW, Australia; 3 Research School of Biology, Australian National University, Canberra, ACT, Australia; 4 University of Essex, UK

**Keywords:** Arabidopsis, biomass vigour, CO_2_ assimilation, early germination, electron transport rate, heterosis, hybrid, leaf development, photosynthesis

## Abstract

*Arabidopsis thaliana* hybrids have similar properties to hybrid crops, with greater biomass relative to the parents. We asked whether the greater biomass was due to increased photosynthetic efficiency per unit leaf area or to overall increased leaf area and increased total photosynthate per plant. We found that photosynthetic parameters (electron transport rate, CO_2_ assimilation rate, chlorophyll content, and chloroplast number) were unchanged on a leaf unit area and unit fresh weight basis between parents and hybrids, indicating that heterosis is not a result of increased photosynthetic efficiency. To investigate the possibility of increased leaf area producing more photosynthate per plant, we studied C24×Landsberg *erecta* (L*er*) hybrids in detail. These hybrids have earlier germination and leaf growth than the parents, leading to a larger leaf area at any point in development of the plant. The developing leaves of the hybrids are significantly larger than those of the parents, with consequent greater production of photosynthate and an increased contribution to heterosis. The set of leaves contributing to heterosis changes as the plant develops; the four most recently emerged leaves make the greatest contribution. As a leaf matures, its contribution to heterosis attenuates. While photosynthesis per unit leaf area is unchanged at any stage of development in the hybrid, leaf area is greater and the amount of photosynthate per plant is increased.

## Teaser

Early germination of hybrids leads to earlier leaf emergence and advanced leaf growth compared with parents, resulting in more photosynthate per plant and heterosis in vegetative growth, with no changes in unit leaf area photosynthesis.

## Introduction

Hybrids often exceed their parents in growth and seed yield; this phenomenon is termed hybrid vigour or heterosis (Shull, 1948). Heterosis is important in crop species including maize and rice (Springer and Stupar, 2007; He *et al.*, 2010) but, although hybrids have been used in agriculture for almost a century, the basis of heterosis is still largely not understood. In our studies on heterosis we have used *Arabidopsis thaliana* hybrids which have properties similar to those of crop species (Groszmann *et al.*, 2014).

Crosses between most Arabidopsis ecotypes produce hybrids that have heterosis in a number of physiological traits, especially in vegetative growth traits such as rosette diameter as well as in seed yield (Meyer *et al.*, 2004; Groszmann *et al.*, 2014; Yang *et al.*, 2017; Wang *et al*., 2019). In both maize and Arabidopsis hybrids, heterosis in vegetative growth is characterized by greater leaf size and a more robust plant (Guo *et al.*, 2010; Groszmann *et al.*, 2014). We asked whether the increased biomass and rosette diameter seen in the Arabidopsis hybrids was the result of increased photosynthetic efficiency or whether the larger photosynthetic leaf area generates the increased growth characteristic of heterotic hybrids. The many hybrids that exhibit heterosis have different levels of heterosis but have similar characteristics. Heterotic hybrids have larger leaves, larger rosette diameter, and greater biomass. We characterized 12 hybrids with respect to growth pattern over development and saw similar trends in development including earlier germination than parents and larger vegetative parameters than the mid-parent value of the parents throughout the life cycle (Groszmann *et al.*, 2014).

Heterosis in biomass is evident in early seedling development (Meyer *et al.*, 2004; Groszmann *et al.*, 2014). Larger leaves and greater growth rely on production and use of photosynthate, raising the possibility that hybrids may have a more efficient photosynthetic process than their parents. Recent findings in Arabidopsis hybrids show that hybrid leaves have an earlier commencement of photosynthesis relative to parents, with earlier expression of photosynthetic genes (Fujimoto *et al.*, 2012; Zhu *et al.*, 2016). The up-regulated genes include chloroplast-targeted genes encoding thylakoid membrane components, the photosynthesis apparatus, and most of the enzymes in the tetrapyrrole and chlorophyll biosynthesis pathways (Fujimoto *et al.*, 2012).

The photosynthetic properties of some maize, rice, and wheat hybrids are similar to those of the parents in the vegetative phase (Mehta and Sarkar, 1992; Baig *et al.*, 1998; Yang *et al.*, 2007; Zhang *et al.*, 2007). In Arabidopsis C24×Columbia-0 (Col) hybrids, the CO_2_ uptake rate was the same in parents and hybrids (Fujimoto *et al.*, 2012). In the present study, photosynthetic parameters of eight Arabidopsis hybrids were compared with those of the corresponding parents to test whether there were any differences. We found that photosynthetic electron transport and carbon metabolism of photosynthesis were identical between hybrids and their parents. The total amount of photosynthate was increased because of the larger area of photosynthetically active tissue in the hybrid leaves. As starch metabolism is critical for growth, we also measured the rate of starch accumulation and turnover in the C24×Landsberg *erecta* (L*er*) hybrids to determine if these properties drove hybrid vigour.

Leaf development involving temporal and spatial coordination of cell division and cell expansion (Donnelly *et al.*, 1999; Kalve *et al.*, 2014) results in leaf size growth caused by altered rates or duration of these two factors (Gonzalez *et al.*, 2012). In Arabidopsis hybrids, heterosis in leaf size is associated with a greater number of cells, and some hybrids have increased cell size (Groszmann *et al.*, 2014). We chose one pair of reciprocal hybrids (C24×L*er*) to study the contribution of individual leaves to hybrid vigour. We chose these hybrids because they had the highest level of heterosis among the different crosses and were representative of the hybrids. We found that during development, biomass heterosis was contributed by changing combinations of differently aged leaves. We found that hybrids germinated earlier than the parents and subsequent leaves emerged earlier. Throughout development hybrids had a greater photosynthate production per plant, contributing to growth and heterosis.

## Materials and methods

### Plant cultivation

*Arabidopsis thaliana* seeds were sterilized with bleach:ethanol solution (2:1) and germinated on sterile agar medium containing Murashige and Skoog (MS) salts supplemented with 3.0% (w/v) sucrose (pH 5.7). The seeds were cold-treated at 4 °C in the dark for 3 d before sowing in controlled-environment chambers (Conviron ATC40 growth chamber, Winnipeg, Canada) under an irradiance of 120 µmol photons m^–2^ s^−1^ (Master TL5 HO 54W/865 SLV/40, Philips Lighting), 21 °C in a 16 h photoperiod. After 10 d in light [10 days after sowing (DAS)], the seedlings were transplanted individually into 65 mm wide×65 mm long×100 mm high square pots containing soil (Debco Seed Raising & Superior Germinating Mix, Debco, Australia). Pots were rotated regularly to minimize position effects. For each experiment, at least three trays were planted.

For short-day experiments, an 8 h photoperiod was used. For doubled-light experiments, plants were grown in a controlled cabinet set to 240 µmol photons m^−2^ s^−1^, under the same temperature and photoperiod as listed above. The light box for half of the shelves in the growth cabinet was covered with a 50% cut-off light filter (Neutral filter 209 0.3ND, LEE Filters, USA) so that the irradiance was reduced to 120 µmol photons m^−2^ s^−1^ without changing the light quality.

### Manual hybridization

Hybrid seeds were produced by manual crossing between ecotypes Col, C24, L*er*, and Wassilewskija (Ws). When at least 5–6 inflorescences had developed on a mature plant it was hand pollinated. Only 3–4 buds per shoot and a maximum of three shoots per plant were pollinated. All lateral shoots, siliques (fruits), excessive buds, and meristems were removed. The same silique-restricting procedure was applied for generating F_1_ parental seeds (Meyer *et al.*, 2004).

### Determination of size, biomass, and growth parameters

Fresh weights of rosettes or leaves were determined immediately after harvest using an electronic semi-microbalance (Type 1872, Sartorius Research, Germany). Comparisons of biomass heterosis were carried out using plants at 19 DAS.

For leaf series measurements, rosette leaves of C24×L*er* plants were cut from the plant and the leaf area was determined at 19 and 30 DAS. All comparisons of heterosis were carried out when plants were in the vegetative phase. C24×L*er* hybrids flowered later than the parents (see Supplementary Table S2 at *JXB* online). Leaf series were obtained by cutting all rosette leaves from the top to the bottom of the rosette (i.e. opposite to the order of leaf emergence). Leaves removed were immediately placed on a Petri dish of MS agar to retain moisture and for the ease of keeping track of the leaf order. Leaf areas were determined from the photos using the image analytic software, ImageJ (National Institute of Health, USA).

The largest leaves of plants at 28 DAS were sampled at ZT7 (ZT: Zeitgeber time, hours in light) for leaf mass per area. A photograph of the leaf was taken upon sampling for leaf area determination. Fresh leaves were wrapped in foil then placed in a 75 °C oven and dried overnight until a constant weight was obtained. The leaf dry weight was determined by subtracting the weight lost overnight from the leaf fresh weight.

Heterosis levels for all growth parameters were defined by the percentage of increase from the mid-parent value (i.e. the average of the parents).

### CO_2_ assimilation

The comparisons of CO_2_ assimilation between hybrids and parents were carried out on plants at 27–29 DAS. To ensure the comparisons were made between similarly developed leaves and to avoid possible variations in photosynthesis at different leaf development stages, the largest leaf of each plant was used. The largest leaf was chosen as it accounts for a large proportion of the rosette biomass (Fig. 6) and its position in the rosette is fully exposed to light with minimum overlap, it is well developed, and the sample was taken before senescence occurs.

All measurements were carried out in a controlled growth room (light cycle 16 h/8 h, 21 °C, 120 µmol photons m^−2^ s^−1^) using a LiCOR infrared gas exchange analyser (6400XT, LI-COR, USA) with a standard 2 cm×3 cm head. To avoid possible effects of time of day on photosynthetic rates, the analyses were carried out within a 6 h window during the day—the first measurement started from 2 h and the last measurement was finished by 8 h after lights on. Due to the large number of plants used and the limited hours for measurement, the experiment took 3 d to complete. The influence of the time of day on both CO_2_ assimilation and chlorophyll fluorescence was tested and we found that there was no difference in measurements carried out at different times of the day. In comparisons of hybrids and parents, the order of samples was randomized so that all replicates were measured at different times in a day and the results were averaged.

The gas exchange chamber air flow rate was 300 µmol s^−1^, leaf temperature 21 °C, actinic light to 120 µmol photons m^−2^ s^−1^ (or 240 µmol photons m^−2^ s^−1^) and the reference [CO_2_] was set to 400 ppm. The relative humidity in the sample cell was controlled within a range between 60% and 70%. The measurement of the *A*/*C*_i_ curve was carried out under a saturating light of 1000 µmol photons m^−2^ s^−1^ with an ascending [CO_2_] of 50, 100, 200, 400, 600, 800, 1000, and 1500 µmol mol^−1^.

### Curve fitting for *A*/*C*_i_ curves

To estimate *V*_cmax_ and *J*, the *A*/C_i_ curves were fitted to the Farquhar–von Caemmerer–Berry model of C_3_ photosynthesis (Farquhar *et al.*, 1980) using the method developed by Sharkey *et al*. (2007). Briefly, data points with *C*_i_ <200 ppm were considered as the Rubisco-limited photosynthesis (designated as ‘1’); the data point with *C*_i_ between 200 ppm and 300 ppm was excluded from the fitting (designated as ‘0’); data points with *C*_i_ >300 ppm were considered as Rubisco bisphosphate (RuBP) regeneration limited (designated as ‘2’); and the data points located at the plateau region of the curve that had a consistent or reduced *A* were considered as the triose phosphate use limited (designated as ‘3’).

### Chlorophyll fluorescence assay

The photon use efficiency of PSII electron transport was determined by using the saturating pulse amplitude modulation method as described by Genty *et al.* (1989) and Schreiber (2004) using a pulse amplitude-modulated (PAM) fluorometer (Closed FluorCam FC 800-C, Photon Systems Instruments, Czech Republic). The seeds were treated and grown on agar plates. The plants were 6 d old when measured.

Briefly, all measurements began with a weak red actinic light (0.1 µmol photon m^−2^ s^−1^) for 5 s. The fluorescence was recorded as the baseline fluorescence, designated as *F*_o_. Next, actinic light (447 nm) was switched on to induce photosynthesis. In order to obtain fluorescence of steady-state photosynthesis, the actinic light was on for 5 min before the fluorescence was recorded, designated as *F*'. The duration of the actinic light incubation was chosen according to a trial measurement carried out on C24 from 3 min to 30 min, showing that *F*' became a constant within 5 min. Right before the actinic light was switched off, a flash (800 ms duration) of saturating light was applied and the fluorescence recorded, designated as *F*_m_'. The fluorescence measurement was completed by applying far-red light for 1 s and the relative minimum fluorescence, *F*_o_', recorded. The prime notation (') used after a fluorescence parameter indicates that the fluorescence parameters are recorded under a continuous actinic light exposure, also known as the light-adapted parameters.

For light–response curves, the illumination cycle described above was repeated with an increasing intensity of actinic light: 30, 200, 330, 440, 550, 640, 720, and 850 µmol photons m^−2^ s^−1^. These light intensities were selected based on a trial light–response curve carried out on the seedlings of C24 and L*er* ecotypes that showed that the light–response curve reaches the plateau phase at ~600 µmol photons m^−2^ s^−1^.

### Calculations of the electron transport rate

The electron transport rate (ETR) was calculated from the light-adapted fluorescence parameters:

$$\begin{array}{l} ETR=PAR\times abs\times b\times \phi_{PSII}=PAR\times abs\times b\times \left[{F_{m}}^{\prime }F^{\prime }\right]/{F_{m}}^{\prime } \end{array}$$

Where ϕ _PSII_ represents the effective quantum yield of PSII; PAR is the incident photosynthetically active radiation; and abs is the leaf absorptance. On average, a healthy leaf of higher plants absorbs ~84% of the incident PAR (Demmig and Bjorkman, 1987). β represents the fraction of the absorbed photons that are subsequently absorbed by PSII. It is generally assumed that the absorbed photons are equally distributed between the two photosystems, PSII and PSI, and a value of β=0.5 was used. The equation for the ETR was calculated as:

$$\begin{array}{l} ETR=PAR\times 0.84\times 0.5\times \left[{F_{m}}^{\prime }F^{\prime }\right]/{F_{m}}^{\prime } \end{array}$$

### Curve fitting for light–response curves

Plotting ETR against an increasing PAR generates a light–response curve. When light intensity is low, the photosynthesis rate is limited by the light, thus the rise of the curve is proportional to the irradiance given. Under high irradiance, ETR is limited by the size of reductant/oxidant pool in the electron transport chain and the ETR reaches a plateau. The light–response curve can be described as the function illustrated below (as described in von Caemmerer, 2000):

$$\begin{array}{l} ETR\;=\frac{\alpha +ETR_{max}-\sqrt{{\left(\alpha +ETR_{max}\right)}^{2}-4\theta \alpha ETR_{max}}}{2\theta } \end{array}$$

where α is the initial slope of the curve; ETR_max_ is the estimated maximum electron transport rate; and θ is an empirical curvature factor. These calculated parameters were fitted for least square deviation.

### Iodine staining for starch granules

Whole rosettes were harvested at the end of the day and the end of the night at 19 DAS. Rosettes were cleared with ethanol to remove pigments before staining with Lugol’s solution (0.1% I_2_ dissolved in 1% KI) overnight. The stained rosettes were rinsed three times with water to remove the excessive Lugol’s solution, transferred to a Petri dish with water, and photographed.

### Starch extraction and quantification

Starch was extracted as described by Ni *et al.* (2009). Pools of 2–3 rosettes were harvested at the end of the night, at 1 h before lights on (ZT15 for long days; ZT7 for short days), and the end of the day, at 1 h before lights off (ZT-1), respectively, at the end of the vegetative phase (19 DAS for long-day plants; 5 weeks for short-day plants). The rosettes were weighed and snap-frozen in liquid nitrogen. Frozen tissue was ground in liquid nitrogen. The homogenate was washed three times with 80% (v/v) ethanol to remove pigments. The starch-containing pellets were collected by centrifugation and re-suspended in absolute ethanol (100 mg FW in 5 ml). The starch suspensions were heated in a boiling water bath for 10 min and vortexed at 2 min intervals to extract starch. A 100 μl aliquot of the homogenate was transferred to 2 ml Eppendorf® Safe-Lock microcentrifuge tubes (Sigma-Aldrich). To reduce the volume of the starch samples, the tubes were placed in a vacuum concentrator (Savant SpeedVac Concentrator, Thermo Scientific) for 3–4 h and the starch slurry was used for further analyses. For each sample analysed, four duplicates of starch slurry were used. Starch content was determined by enzymatic quantification. The Total Starch Assay Kit (Amyloglucosidase/α-Amylase method, Megazyme, Ireland) was used and the manufacturer’s protocol was followed.

### Chlorophyll quantification

Four-week-old plants were weighed and chlorophylls were extracted by homogenizing rosettes in liquid nitrogen using a pestle and mortar. The tissue powder was dissolved in 95% (v/v) ethanol. The tissue debris was spun down by centrifugation and the chlorophyll-containing supernatants were kept on ice, in the dark to prevent degradation of chlorophylls. The chlorophyll-containing supernatants were used for absorbance determination at wavelengths 664.2 nm and 648.6 nm as described by Porra *et al.* (1989). The Chl *a* and Chl *b* contents were determined using the formulae described by (Lichtenthaler and Buschmann, 2001):

$$\begin{array}{l} \begin{array}{ll} & Chl\;a\;(\;\mu \;g\;ml^{1})=13.36\times A^{664}5.19\times A^{648}\\ & Chl\;b\;(\;\mu \;g\;ml^{1})=27.43\times A^{648}8.12\times A^{664} \end{array} \end{array}$$

### Chloroplast number per mesophyll cell

The number of chloroplasts per mesophyll cell was determined following the protocol described by Okazaki *et al.* (2009). Briefly, cotyledons or leaves were fixed with 3.5% glutaraldehyde for 2 h and de-calcified with 0.1 M Na_2_-EDTA (pH 9.0) at 50 °C for 15 min. Single mesophyll cells were isolated by gently pressing the fixed tissue between a glass slide and a coverslip using the eraser end of a pencil. The microscopic images of the isolated, intact mesophyll cells were taken under differential interference contrast (DIC) optics using an optical microscope (Axio Imager, Zeiss, USA) and the number of chloroplasts per cell was counted. The number of chloroplasts per cell was then divided by the cell area determined by the image analytical software ImageJ (National Institutes of Health, USA).

### Determination of leaf cross-section thickness

The measurements of leaf thickness were carried out using cross-sections prepared from the centre of the leaf lamina. The microscopic images of leaf sections were taken at the regions near the midvein, at positions without trichomes.

### Statistical analysis

Statistical comparisons between hybrids and the parents (or the average level of the parents) as well as all other comparisons were carried out using the Fit An Analysis Of Variance Model function (function ‘aov’, R Stats Package ‘stats v3.4.1’) in conjunction with the compute Tukey honest significant differences (function ‘TukeyHSD’, R Stats Package ‘stats v3.4.1’).

## Results

### Photosynthetic parameters are the same in hybrids and parents

**Fig. 1.**
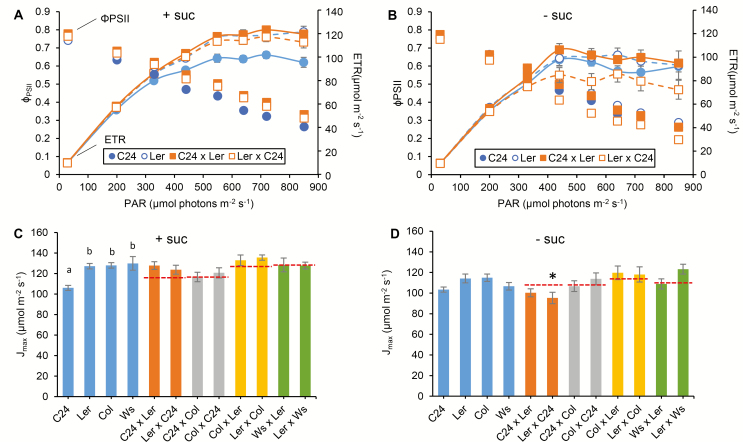
The light–response curves of the quantum yield of PSII (ϕ _PSII_) and the electron transport rate (ETR) of the 6 DAS C24×L*er* hybrid seedlings grown on MS medium either (A) with or (B) without 3% sucrose. The chlorophyll fluorescence parameters were analysed and used to calculate ϕ _PSII_ as described by Genty *et al.* (1989) using a pulse amplitude-modulated fluorometer (Closed FC 800-C, PSI). Each data point represents the average and SE of *n*≥5. The ETR was calculated by ETR=ϕ _PSII_×PAR×0.5×0.84, as described in Edwards and Baker (1993). (C and D) The maximum electron transport rates (*J*_max_) calculated by fitting the ETR curves of parental lines and hybrids grown either with or without sucrose to the C_3_ photosynthesis model. See Supplementary Fig. S1 for the chlorophyll fluorescence light–response curves of other hybrids used in this experiment. Statistical comparisons among parents are shown; datasets with different letters are significantly different from the others (ANOVA, *P*<0.05). Asterisks indicate a significant difference between the hybrid and the better parent (ANOVA, *P*<0.05). No significant differences were found between hybrids and the mid-parent value (indicated by red dashed lines) and between hybrids and the lower parent (ANOVA, *P*>0.05).

To determine whether the capacity of photosynthetic processes was different in the hybrids from that in the parents, F_1_ hybrids and parents were analysed by chlorophyll fluorescence (ϕ _PSII_ and photosynthetic ETR) and gas exchange (CO_2_ assimilation rate). Young seedlings (6 DAS) of eight hybrids derived from four ecotypes, C24, L*er*, Col, and Ws and their parents were analysed for ETR estimated by PSII chlorophyll fluorescence under increasing light intensities (Fig. 1; Supplementary Fig. S1). To avoid any possible inhibition of photosynthesis by exogenous sucrose in the culture medium (Lobo *et al.*, 2015), a sucrose-free condition was included (Fig. 1; Supplementary Fig. S1). The maximum ETRs (*J*_max_) of all hybrids and parental lines were estimated from the ETR curves against light intensity (Fig. 1C, D). No significant increase in *J*_max_ was found in hybrids relative to the parents (ANOVA, *P*>0.05), showing that hybrids and parents do not differ significantly in the molecular properties of the light reaction component of photosynthesis.

**Fig. 2.**
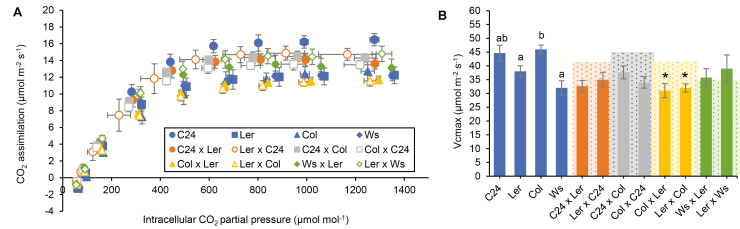
The CO_2_ gas exchange of 3-week-old hybrids compared with parents. The plants were sown on MS solid medium before being transferred into soil at 10 d old. Plants were grown under a light cycle of 16 h/8 h, in a light intensity of 120 µmol photons m^−2^s^−1^, at 21 °C. (A) The net CO_2_ assimilation rate of the hybrids and parents was measured under an increasing partial pressure of atmospheric CO_2_ under a saturating irradiance at 1000 µmol m^−2^ s^−1^ using a gas exchange analyser (LI-6400XT, LICOR). The *x*-axis shows the intercellular CO_2_ partial pressure. The analyses were carried out on the largest leaf on the rosette of each plant measured. Each data point represents the average and SE of *n*≥3. (B) Comparison of the maximum rate of Rubisco carboxylation (*V*_cmax_) between hybrids and the parents. The curve fitting result using the dataset from (A). The curve fitting was carried out by fitting the dataset from (A) to the C_3_ photosynthesis model (Farquhar *et al.*, 1980). Each data point represents the average and SE of *n*=3. Different letters above the columns represent a significant difference between the parents (ANOVA, *P*<0.05). Asterisks represent significant differences between the hybrid and the better parent (ANOVA, *P*<0.05). No significant differences were found between hybrids and the lower parents, and between the hybrids and the growth under the average value of the parents (indicated by red dashed lines; ANOVA, *P*>0.05).

Three-week-old hybrids were analysed for CO_2_ assimilation rate (*A*) per unit leaf area under increasing CO_2_ partial pressure (*C*_i_) (Fig. 2A). Modelling of these data (Farquhar *et al.*, 1980) from all eight hybrids showed a maximum velocity of Rubisco carboxylation (*V*_cmax_) within the range of that of the corresponding parents (Fig. 2B). These results indicate that hybrids are not significantly different from parents in photosynthetic carbon metabolism parameters on a unit leaf area basis.

### Starch production and catabolism

**Fig. 3.**
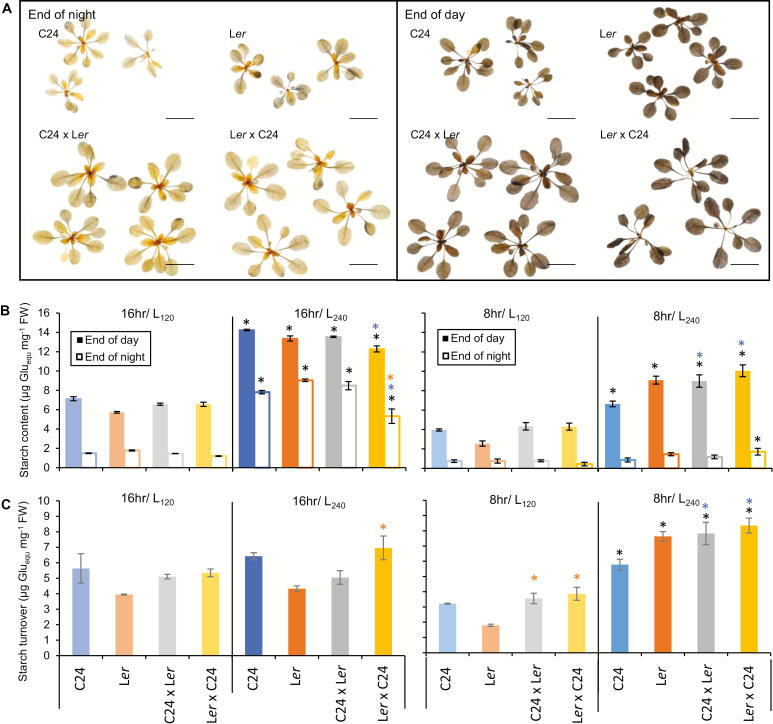
Qualitative analyses of starch turnover under standard conditions. (A) Arabidopsis seedlings were grown under a light cycle of 16 h light/8 h dark, in a light intensity of 120 µmol m^−2^ s^−1^, at 21 °C. Rosettes of 21 DAS seedlings were harvested at the end of the night and the end of the day, respectively, and stained with Lugol’s iodine solution (0.1%). Dark purple stain indicates the presence of starch granules. Scale bars=2 cm. (B) Quantitative analyses of starch content of C24×L*er* grown in long (16 h/8 h) or short (8 h/16 h) photoperiods under standard (120 µmol m^−2^ s^−1^) or doubled (240 µmol m^−2^ s^−1^; designated as L_240_) light intensity. Starch content is presented as the equivalent amount of glucose. Data shown were the average and SE of three technical replicates from a pool of *n*=4–6 rosettes. For long photoperiods, 21 DAS plants were used, whereas 40 DAS plants were used for short photoperiods. (C) The unit biomass starch turnover per night. This was calculated by subtracting the average starch content at the end of the night from that at the end of the day (SE, *n*=4–6). End of the day, 1 h before lights off; end of the night, 1 h before lights on. Asterisks indicate significant differences from growth under the standard light conditions (ANOVA; *P<0.05). Significant differences between the hybrid and the parents are represented by asterisks in colour (blue, C24; orange, L*er*; ANOVA, *P<0.05).

Starch is one of the main products of photosynthesis, and starch catabolism is an integral part of the use of photosynthate for growth. We compared both starch production and starch catabolism in parents and hybrids to determine effects on heterosis. Starch content in leaves of 16 h/120 µE-grown C24×L*er* hybrids and parents was high at dusk and low at dawn (Fig. 3A), typical of the diurnal starch turnover in Arabidopsis (Smith, 2012). Under long photoperiods, the 16 h/120 µE-grown C24×L*er* hybrid seedlings contained 6.5 µg mg^–1^ FW of starch at the end of the day, similar to the average of that of the parents (Fig. 3B). By dawn, both hybrids and parents had metabolized >75% of the accumulated starch. The starch turnover in the 21 DAS 16 h/120 µmol photon m^−2^ s^−1^-grown C24×L*er* hybrids was just above 5 µg mg^−1^ FW, slightly greater than the average of that of the parents but not greater than that of the C24 parent (Fig. 3C).

The doubling of the photon density approximately doubled starch production per day. At the end of the day, the 16 h/240 µE-grown hybrids and parents contained an average of 13±1.6 µg mg^−1^ FW of starch (Fig. 3B, C). Although starch biosynthesis was greater under higher irradiance, the end of the night starch content in these 16 h/240 µE-grown plants was approximately half that at the end of the previous day (Fig. 3B), which means that the 16 h/240 µE-grown plants were not able to fully remobilize the increased amount of starch accumulated under the doubled irradiance conditions. Starch turnover in the longer photoperiods was possibly limited by the remobilization rate, as the total consumption per night was the same in 120 µE- and 240 µE-grown plants. Under both light levels tested, the starch turnover of hybrids was comparable with that of the parents, but the total amount of starch produced was greater in the hybrid leaves with their larger area.

In short photoperiods, starch production was, as expected, less than that in the same irradiance in long photoperiods. The 8 h/240 µE-grown plants had 8±0.7 µg mg^−1^ FW of starch at the end of the day, about twice that with 8 h/120 µE (Fig. 3B). The short-day plants grown under either 120 µE or 240 µE utilized >80% of the accumulated starch during the night (Fig. 3B), a greater amount than the 8 h/120 µE-grown plants on a unit biomass basis. These results showed that increasing photon density in the day increases starch production and that longer nights are required for a greater level of starch turnover and increased duration of growth. The C24×L*er* reciprocal hybrids had a total starch consumption no greater than the better parent on a unit biomass basis per night under all light regimes tested (*P*>0.05).

### Leaf anatomy of parents and hybrids

To determine whether the photosynthetic apparatus in hybrid leaves is altered, leaves from C24×L*er* hybrids were analysed for possible alterations in anatomy and the content of chlorophyll and chloroplasts relative to parents. All leaves responded to higher light with increased leaf thickness. The hybrids had a leaf mass per unit area at levels within the range of the corresponding parents, as did four other combinations of hybrids (Supplementary Fig. S2A–C). These data show that the leaf anatomy of hybrids did not differ from that of the parents. C24×L*er* hybrids and parents showed a similar chloroplast density per mesophyll cell, with a positive correlation between the number of chloroplasts per cell and cell area (regression coefficient >0.9) (Supplementary Fig. S3). The chlorophyll content in the leaves of hybrids was not different from that of the parents on a unit leaf area basis (Supplementary Table S1), suggesting that the photosynthetic capacity of hybrid leaves is similar to that of the parental leaves on a per unit fresh weight or on a chlorophyll concentration basis. The total photosynthetic area of the larger hybrid leaves was greater than that of the parental leaves, enabling the hybrids to produce more photosynthate than the parents (Groszmann *et al.*, 2014). The leaves of parents and hybrids had comparable anatomy under different conditions of irradiance (Supplementary Fig. S2).

### Higher irradiance in 8 h photoperiods increases biomass heterosis in C24×L*er* hybrids

**Fig. 4.**
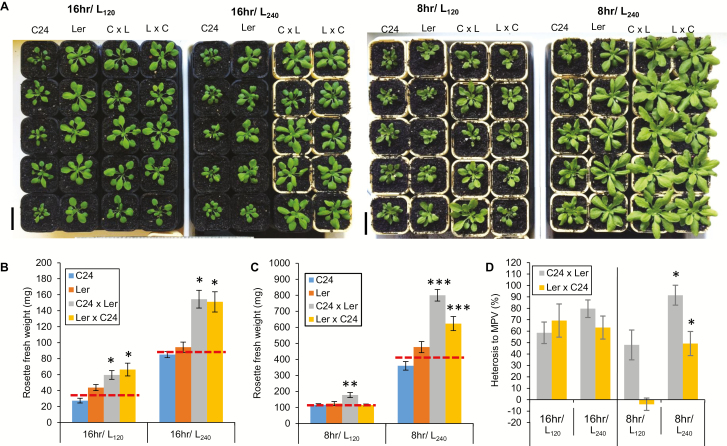
C24×L*er* hybrid seedlings grown under a photon density of 120 µmol m^−2^ s^−1^ (L_120_) or 240 µmol m^−2^ s^−1^ (L_240_) under short (8 h) or long (16 h) photoperiods. Arabidopsis seedlings were grown under 120 µmol m^−2^ s^−1^ for 10 DAS before half of the seedlings were transferred into 240 µmol photons m^−2^ s^−1^. The 18 DAS 240 µmol photons m^−2^ s^−1^-grown plants were assessed for heterosis in vegetative biomass and compared with the 120 µmol photons m^−2^ s^−1^-grown seedlings. (A) The images of the 35 DAS and 21 DAS C24×L*er* grown under 8 h and 16 h photoperiods, respectively. (B and C) Measurements of rosette biomass. Data presented are the average and SE of *n*>15 from 2–3 experiments. Asterisks indicate that the hybrids were significantly different from the average levels of the parents (red dashed lines) (ANOVA, *P<0.05, **P<0.01, ***P<0.001). (D) Heterosis levels of hybrids presented as a percentage increase from the average levels of the parents. Statistical comparisons were carried out between and within hybrids in different light conditions. The asterisks indicate a significant difference from the control light under the same photoperiod (ANOVA, *P*<0.05).

Meyer *et al.* (2004) found that doubling growth irradiance increases heterosis levels in Col×C24, Cvi×C24, and RLD×C24 Arabidopsis hybrids. We used the C24×L*er* hybrid system for detailed characterization of carbohydrate production and turnover as well as for assessing the contribution of individual leaves to heterosis during development. The C24×L*er* hybrids and parents grown under an irradiance of 240 µmol photon m^−2^ s^−1^ (240 µE), namely at twice the control photon density (120 µE), were analysed for biomass heterosis. Under either long (16 h) or short (8 h) photoperiods, 240 µE-grown plants had shorter petioles and more compact rosettes than 120 µE-grown plants (Fig. 4A). The heterosis levels of the 16 h/240 µE hybrids were not significantly different from that in 16 h/120 µE (ANOVA, *P*>0.05), both reciprocal C24×L*er* hybrids showing a 60–70% greater biomass than the average of the parents (Fig. 4D) in agreement with the results of Meyer *et al*. (2004) who did not find any significant difference between 240 µmol m^−2^ s^−1^ and 120 µmol m^−2^ s^−1^ in C24×L*er* hybrids. Biomass of both parents and hybrids grown at 240 µE was more than twice that of the 120 µE-grown plants (Fig. 4B).

The rosette fresh weight (without roots) of short-day-grown plants was determined at 5 weeks (35 DAS) after sowing. This time point was chosen as the plants were at a similar developmental stage as the 3-week-old long-day plants (i.e. before L*er* flowered; Supplementary Table S2). The 35 DAS, 8 h/120 µE-grown C24×L*er* hybrid had an average fresh weight of ~180 mg while its reciprocal hybrid and parents had an average rosette fresh weight of ~100 mg (Fig. 4C). When grown under 8 h/240 µE, the 35 DAS C24×L*er* and the reciprocal hybrid showed a significant increase in heterosis from 48% to 98% and from 0 to 50% (ANOVA, *P*<0.05), respectively, compared with the average biomass of the parents (Fig. 4D).The difference between reciprocal hybrids could result from a differing sensitivity of L*er* to low light growth conditions.

An increase in photon density affects photosynthesis directly by changing the amount of energy available for electron transport and carbon fixation, producing more sugars and starch. Gas exchange analyses of the plants grown in long photoperiods (16 h) showed no significant difference in the photosynthetic parameters, *V*_cmax_ or *J*, between hybrids and parents when measured under saturating light (ANOVA, *P*>0.05) (Supplementary Fig. S4), showing that there was no difference in the unit leaf area photosynthetic capacity between 120 µE- and 240 µE-grown plants. These results indicate that the biochemical capacity of photosynthesis in hybrids was similar to that of the parents, regardless of the growth irradiance. As photosynthesis is light dependent and the greater photon density increases photosynthate production, the 240 µE-grown plants assimilated more CO_2_ per day than the 120 µE-grown plants and had thicker leaves (<200 µm versus 250 µm) compared with the leaves of the 120 µE-grown plants due to increased cell volume and cell number (Supplementary Fig. S3A, B). The unit leaf area photosynthesis in the thicker leaves of the 240 µE plants was significantly greater than that in the 120 µE-grown plants, due to an increased number of spongy mesophyll cells. Under both photon densities, the thickness of hybrid leaves and their chlorophyll contents were not significantly greater than those of the L*er* parent (Supplementary Fig. S2; Supplementary Table S1). These results indicate that the capacity for photosynthate production of hybrid plants per unit leaf area was not greater than that of the parents.

### Leaf size and contributions to heterosis

**Fig. 5.**
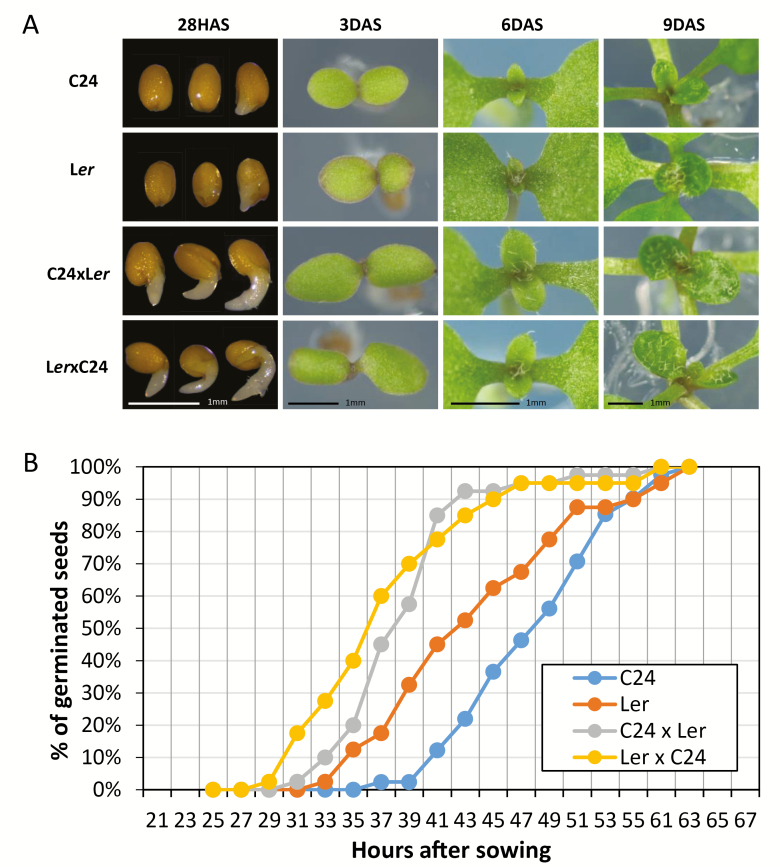
Earlier germination and leaf emergence in C24×L*er* hybrids relative to parents. (A) Photographs of parents and reciprocal hybrids at 28 h after sowing (HAS), 3, 6, and 9 DAS. (B) Germination of C24×L*er* hybrids and parents (see also Zhu *et al.*, 2016). Germination was scored as the first visible sign of root emergence.

The earlier growth of hybrid leaves compared with the leaves of the parents is associated with the significantly earlier germination of the hybrids relative to the parents by 8–10 h (Fig. 5; Zhu *et al.*, 2016). In agreement with Meyer *et al*. (2012) and Wang *et al*. (2019), we found that the cotyledons were larger in hybrids at 3 DAS. At 6 DAS, the first true leaf had emerged earlier in the hybrids; by 9 DAS, the second leaf had emerged and was larger in the hybrids than in the parents. The C24×Col hybrid also showed earlier germination than the parents, driving the increased leaf area and total photosynthate accumulation in C24×Col hybrids (Fujimoto *et al.*, 2012). Earlier germination was observed in the Col×L*er* and Ws×L*er* hybrids compared with their parents (Wang *et al.*, 2019). In each hybrid, the early germination resulted in earlier greening of cotyledons and young leaves, showing an earlier onset of photosynthesis in the larger leaves.

**Fig. 6.**
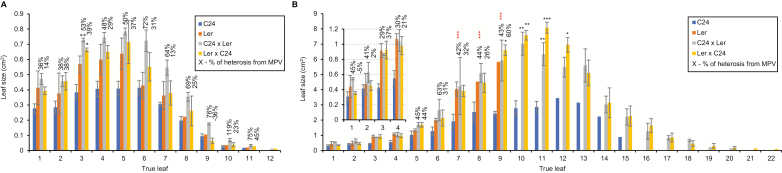
Measurements of the true leaves of C24×L*er* hybrids at (A) 19 DAS and (B) 30 DAS. True leaves on a rosette were numbered in the order of their positions in the rosette, from the bottom to the top of the rosette (i.e. the order of emergence from the meristem). In each line, three seedlings were examined. Error bars represent the SE of *n*=3 plants. Columns without error bars represent the average of data from *n*<3. Black asterisks indicate a significant difference from the better parent; red asterisks of 30 DAS leaves indicate a significant difference in the heterosis levels from the corresponding leaves at 19 DAS (ANOVA; *P<0.05, **P<0.01, ***P<0.001). Statistical comparison was carried out only on datasets containing three biological replicates.

The leaves of the C24×L*er* hybrids were removed at 19 and 30 DAS in the opposite order of emergence and the leaf area was measured and compared with that of the corresponding parent leaves (Fig. 6). The area of each hybrid leaf was greater than the average size of the corresponding parent leaf (Fig. 6). The hybrids show significant (*P*<0.001) changes in leaf area heterosis levels in successive leaves which contribute differentially to biomass heterosis on a whole-plant basis over the early growth period up to 30 DAS. In C24×L*er* hybrid seedlings at 19 DAS, leaves 1 and 2 had a heterosis level of ~40%; 40–60% heterosis in leaves 3–6; and >60% in leaves beyond position 6 (Fig. 6A). Lower levels of heterosis were found in the leaves of the reciprocal L*er*×C24 hybrid (Fig. 6A).

**Fig. 7.**
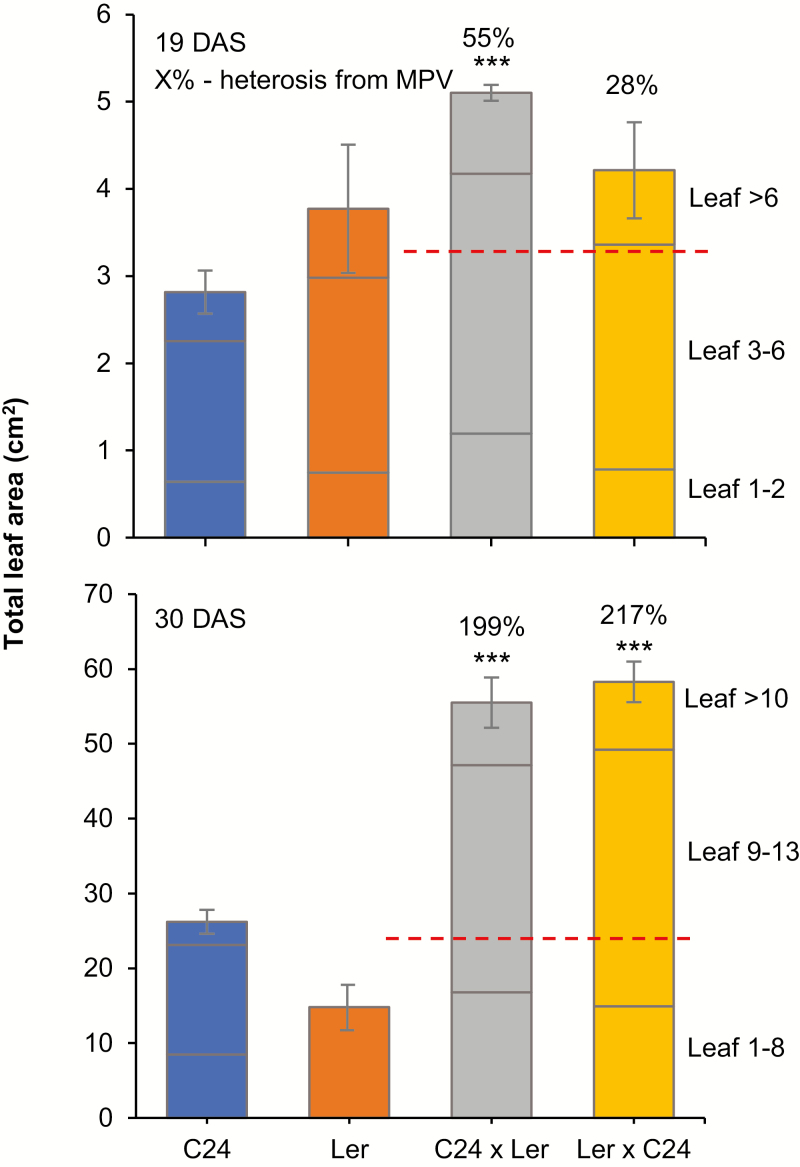
Relative contribution of leaf sets to the whole-plant biomass. Data presented are the average and SE of the sum of the leaf area per rosette, using the dataset from Fig. 6. Each data point was divided into three fractions: indicating the relative contribution of the largest leaves and the rest of the leaves in a seedling, in the order of their relative positions in a rosette. Percentages above columns represent the percentage of heterosis relative to the mid-parent value (red dashed lines). Asterisks indicate significant differences from the average level of the parents. *P<0.05, **P<0.001, ***P<0.001 (ANOVA). MPV, mid-parent value.

At 19 DAS, the C24×L*er* hybrid and the reciprocal L*er*×C24 hybrids had a total leaf area 55% and 28% greater than the average of that of the parents, respectively (Fig. 7). At 19 DAS, plants of C24×L*er* hybrids and parents contained 11–12 leaves per rosette (Fig. 6A). In the hybrid seedlings at 19 DAS, the largest leaves, 3–6 (Fig. 6A), were the primary contributors to heterosis in growth as they accounted for ~60% of the total leaf area at this stage of development (Fig. 7, top panel). The newly formed leaves 7–12 made relatively little contribution to heterosis at 19 DAS as they only accounted for a small fraction of the total biomass (Fig. 7, top panel). These newly developed leaves in turn contributed more to hybrid vigour in subsequent stages.

At 30 DAS, both the C24×L*er* reciprocal hybrids showed increased heterosis in total leaf area, which was up to 200% greater than the average of that of the parents (Fig. 7, bottom panel). The increasing difference between hybrids and parents in total leaf area at this developmental stage was mainly due to the number of new leaves being produced in hybrid rosettes: at 30 DAS, hybrid seedlings contained an average of 20 leaves per rosette (Fig. 6B; Supplementary Table S2). The largest leaves were leaves 9–13 (Fig. 6B), accounting for >50% of the total leaf area (Fig. 7, bottom panel). Those leaves were not present at 30 DAS in L*er* seedlings (Fig. 6B) because the early flowering ecotype L*er* ceased to produce new rosette leaves shortly after 19 DAS and leaves 9–12 became cauline leaves. C24 continued to produce rosette leaves until flowering at 31 DAS and hybrids until 39 DAS (Supplementary Table S2). At 30 DAS, despite having fewer leaves per rosette, leaf size of the L*er* plants equalled that of the hybrids in the nine leaves present before flowering (Fig. 6B). When fully expanded, these L*er* leaves reached the same final size as hybrid leaves, whereas C24 leaves were always smaller. The final size of each hybrid leaf is reached earlier than that of comparable parent leaves, probably due to an increased rate of cell expansion and/or an earlier commencement of the cell expansion phase, as suggested by the greater cell size observed in young hybrid leaves (Supplementary Table S3). The heterosis levels of leaves 1–8 became less marked at 30 DAS (Fig. 5B). By 30 DAS, C24 plants were not producing as many new leaves as the hybrids and contained an average of 14 leaves per rosette (Supplementary Table S2). During early stages of seedling development, the rate of leaf appearance and the succession of newly developed leaves, larger in the hybrids than in the parents, contributed to heterosis. Later in development, hybrid vigour is primarily a result of increased leaf number due to delayed flowering of the hybrids. The larger size of Arabidopsis hybrid leaves relative to parents at later stages of development is a combination of a greater number of cells per leaf and increased cell size (Supplementary Table S3), with different proportions in different hybrid combinations (Groszmann *et al.*, 2014). Hybrid leaves have a greater number of palisade mesophyll cells than the parental leaves (Supplementary Fig. S5). The larger leaves produce more total photosynthate and contribute more to heterosis (Wang *et al.*, 2019).

## Discussion

### No changes in basic photosynthetic reactions in hybrids

Photosynthetic parameters (photosynthetic electron transport and carbon metabolism) were unchanged in the hybrids: the reactions are essentially the same in parents and hybrids. Both the light and dark reactions were measured and show that the light-capturing efficiency of PSII and the interaction with PSI, including the CO_2_ assimilation rate, remain unchanged between parents and hybrids.

We conclude that changes in photosynthetic parameters are not responsible for heterosis. Photoperiod and irradiance do influence heterosis. We have shown that both the amount of photosynthate assimilated and turnover of photosynthate occurring over a 24 h period must be considered. It has been suggested that the circadian clock plays a role in heterosis in Arabidopsis C24×Col hybrids by up-regulating photosynthesis and increasing starch production (Ni *et al.*, 2009); however, our findings in C24×L*er* hybrids showed no changes in photosynthesis processes or in starch turnover relative to parents. We found that shorter nights increased the capacity of hybrids to turn over starch in the dark period and increase heterosis (see Fig. 4).

### Rate of leaf development and leaf size contribute to biomass heterosis

In a C24×L*er* hybrid leaf series, biomass is mainly contributed by the largest 4–5 leaves present at any one time (Fig. 6). Leaf development has four phases: an initiation phase; a general cell division phase; a transition phase; and a cell expansion phase (Gonzalez *et al.*, 2012). Changes in the rate or duration of any of the phases result in changes in leaf size due to altered cell size and/or cell number (Gonzalez *et al.*, 2012).

C24×L*er* hybrids have at least one leaf more than parents from 15 DAS onwards (*P*<0.05, Supplementary Table S2). The increase in hybrid leaf number is attributable to the earlier emergence of successive leaves. After initiation from the shoot meristem, early leaf growth is mediated by cell division, and cell size is generally unchanged (Donnelly *et al.*, 1999). Transcriptome analyses of hybrids have shown up-regulated auxin biosynthesis and signaling pathways (Blasing *et al*., 2005; Groszmann *et al.*, 2015), suggesting a role for auxin in the generation of hybrid vigour. In hybrids there is earlier up-regulation of auxin pathways in the germinating seed and in the leaves, which emerged earlier than in parents and accounted for the head start in growth of the hybrids (Wang *et al*., 2019).

As a leaf grows, cell division ceases and leaf growth is mediated by cell expansion. At different developmental stages, C24×L*er* leaves show significant heterosis in leaf size attributable to larger cell size compared with the parental leaves (Supplementary Table S3). Hybrid leaves reach their final size faster than the parental leaves. When fully expanded, L*er* catches up with the C24×L*er* hybrid in leaf size, and the differences in the palisade mesophyll cell size between the hybrid and the parent become less marked compared with that in the younger leaves (Fig. 6; Supplementary Table S3).

The C24×L*er* hybrid germinated earlier than the parents, resulting in earlier emergence of cotyledons and leaves and a larger leaf area and more total photosynthate at all points of development. This is true for different hybrid combinations (Wang *et al*., 2019). Earlier germination may be a general characteristic of hybrids as it also occurs in crop hybrids such as maize (Sarkissian *et al.*, 1964). Upon germination, plants are exposed to an important environmental stimulus, light, which stimulates photosynthetic gene activity and photosynthesis.

### Carbohydrate availability and biomass heterosis

Transcriptome analyses have shown an earlier up-regulation of genes in photosynthetic pathways in seedlings of C24×Col and C24×L*er* hybrids compared with the parents (3 versus 5 DAS) (Fujimoto *et al.*, 2012; Zhu *et al.*, 2016). Analyses of the relative ETR and CO_2_ assimilation rate in a cohort of eight hybrids from four Arabidopsis ecotypes at different stages during the vegetative phase showed that hybrids have a photosynthetic capacity equal to that of the parents on a unit leaf area basis (Figs 1, 2). The C24×L*er* hybrid has a chlorophyll content per unit area in the leaves similar to the parents (Supplementary Table S1) and a starch turnover not greater than the better parent (Fig. 3). These results indicate that the photosynthesis processes in hybrids are not different from those in the parents but that the larger leaf sizes of the hybrid result in a significantly greater amount of photosynthate than the parents. The earlier germination and onset of leaf growth are important precursors for hybrid vigour.

When grown under short photoperiods (8 h), the C24×L*er* hybrids that were grown under 240 µE of light had a heterosis level twice as great as when grown under 120 µE (Fig. 4C). Under the short photoperiods, plants that were grown under 240 µE had a starch turnover significantly greater than that under 120 µE (Fig. 4). Regardless of the light regime (long/short photoperiods, with or without doubling of photon density), the C24×L*er* hybrids had similar photosynthetic capacity per unit leaf area (Supplementary Fig. S4), similar unit biomass chlorophyll content (Supplementary Table S1), similar leaf anatomy (Supplementary Fig. S3), and similar unit biomass starch turnover to the parents (Fig. 3). These results indicate that heterotic growth is dependent on the carbohydrates produced by photosynthesis and on the ability of leaves to utilize stored carbon during the dark period and allocate this carbohydrate to biomass. The importance of carbohydrate utilization is exemplified in the comparison of heterosis in hybrids grown in short days (8 h) under 240 µE with those grown in long days (16 h) at 120 µE. Starch turnover, enhanced in short photoperiods, contributes to growth and the level of heterosis (Blasing *et al*., 2005; Graf *et al.*, 2010).

Plants grown under these two conditions have the same ‘photon dose’, namely the same capacity for net carbon gain, but in the case of the plants under short days, rosette biomass was 10- to 15-fold higher (~40 mg versus 400 mg, Fig. 4B, C). Heterosis in C24×L*er* hybrids was almost 2-fold greater under short days than under long days with the same diurnal photon dose (Fig. 4D), similar to the result under long photoperiods for C24×Col hybrids (Meyer *et al.*, 2004). The importance of day length for management of leaf starch turnover is shown in Fig. 4. Rosettes of C24×L*er* grown under 240 µE and long days accumulated starch which could not be fully metabolized during the short night (Fig. 3B), as happens in plants grown in very long photoperiods (Sulpice *et al.*, 2014).

The vegetative biomass of a plant is the net result of carbon gain in source tissues, mainly the leaves, carbon loss in respiration, and carbon allocation to other sink tissues such as shoot meristem and roots. Although the unit area/biomass photosynthesis is the same in hybrids and parents, the hybrid plant has significantly larger leaves as a result of the earlier germination and the earlier emergence of each leaf, as shown by the greater leaf number at any time in development (Supplementary Table S3). This advantage in hybrid growth is cumulative during development. Different sets of leaves contribute to heterosis at different times of development. The results presented in our study show that a more efficient photosynthetic process in hybrids, on a leaf area or leaf biomass basis, is unlikely to be responsible for increased growth relative to parents. An increased rate of leaf appearance and expansion suggests a fundamental difference in response of hybrids to photosynthate accumulated during the photoperiod. While the mechanistic basis of this process is not fully described, sugar and hormone signaling controlling leaf emergence and expansion could be the basic factors for the enhanced growth in hybrids. Supply of photosynthate is critical for the level of heterosis, and the photosynthate production due to larger leaves is enhanced in hybrids.

In conclusion, photosynthetic efficiency per unit leaf area does not drive heterosis but faster development and increased leaf area result in higher photosynthate production per plant in hybrids, compounding and driving higher biomass generating heterosis.

## Supplementary data

Supplementary data are available at *JXB* online.

Fig. S1. The light–response curves of the quantum yield of PSII (ϕ _PSII_) and the electron transport rate (ETR) of the 6 DAS hybrid seedlings grown on MS medium either with or without 3% sucrose.

Fig. S2. Comparisons of leaf morphology between hybrids and parents.

Fig. S3. Comparisons of chloroplast content between the C24×L*er* hybrids and parents.

Fig. S4. Photosynthetic parameters of 240 µmol m^−2^ s^−1^-grown C24×L*er* hybrids and parents.

Fig. S5. Cellular basis of leaf size differences between hybrids and parents. Leaves 3 and 4 were analysed as a pair and the results were averaged.

Table S1. Chlorophyll content of the 4-week-old C24×L*er* hybrid seedlings grown under a 16 h photoperiod.

Table S2. Leaf number and flowering time of C24×L*er* hybrids and parents.

Table S3. Measurements of palisade mesophyll cells of leaves 3/4 of 19 DAS and 28 DAS and leaves 5/6 of 19 DAS C24×L*er* hybrid plants compared with the parents.

eraa006_suppl_Supplementary_Figures_S1-S5_and_Tables_S1-S3

## Abbreviations

A: net CO_2_ assimilation
Col: Columbia-0
*C*_i_: partial pressure of intercellular CO_2_
DAS: days after sowing
ETR: electron transport rate
*J*_max_: maximum electron transport rate
L*er*: Landsberg *erecta*
MS: Murashige and Skoog
PAR: photosynthetically active radiation
ϕ _PSII_: quantum yield of PSII
*V*_cmax_: maximum velocity of Rubisco carboxylation
Ws: Wassilewskija
