# Supplementary material for: Leaf growth in early development is key to biomass heterosis in Arabidopsis
Source: J Exp Bot. 2020 Jan 21;71(8):2439–50. doi: 10.1093/jxb/eraa006 (PMC7178430; doi:10.1093/jxb/eraa006)
Supplement: eraa006_suppl_Supplementary_Figures_S1-S5_and_Tables_S1-S3 [file eraa006_suppl_supplementary_figures_s1-s5_and_tables_s1-s3.pdf]

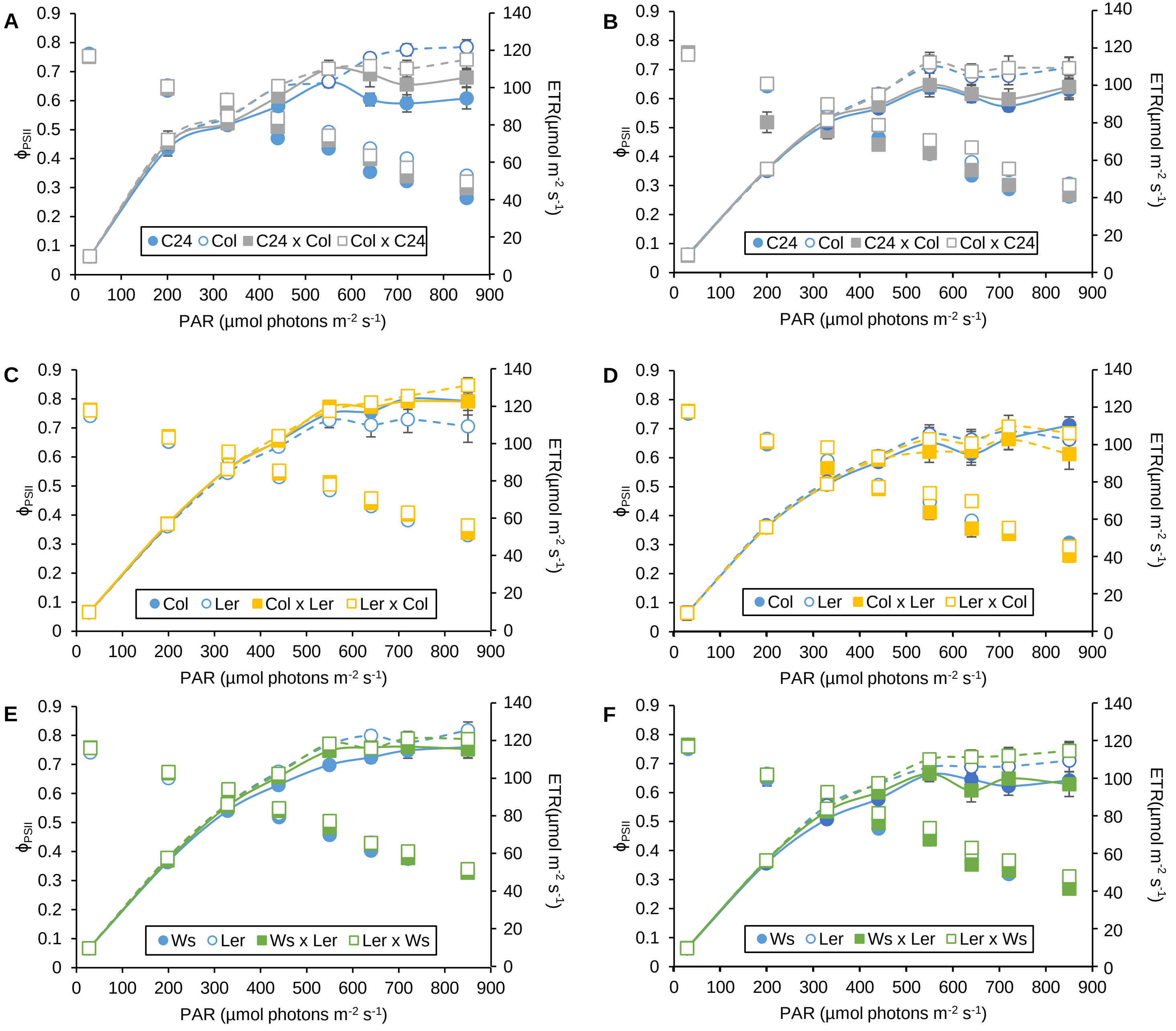

Supplementary Figure 1. The light-response curves of the quantum yield of photosystem II (ϕ_{PSII}) and the electron transport rate (ETR) of the 6 DAS hybrid seedlings grown on MS medium either (A, C, E) with or (B, D, F) without 3% sucrose. The chlorophyll fluorescence parameters were analysed and used to calculate ϕ_{PSII} as described by Genty *et al.* (1989) using a pulse amplitude modulated fluorometer (Closed FC 800-C, PSI). Each data point represents the average and SE of $n \geq 5$. The ETR was calculated by ETR = $\phi_{PSII} \times \text{PAR} \times 0.5 \times 0.84$, as described in Edwards and Baker (1993).

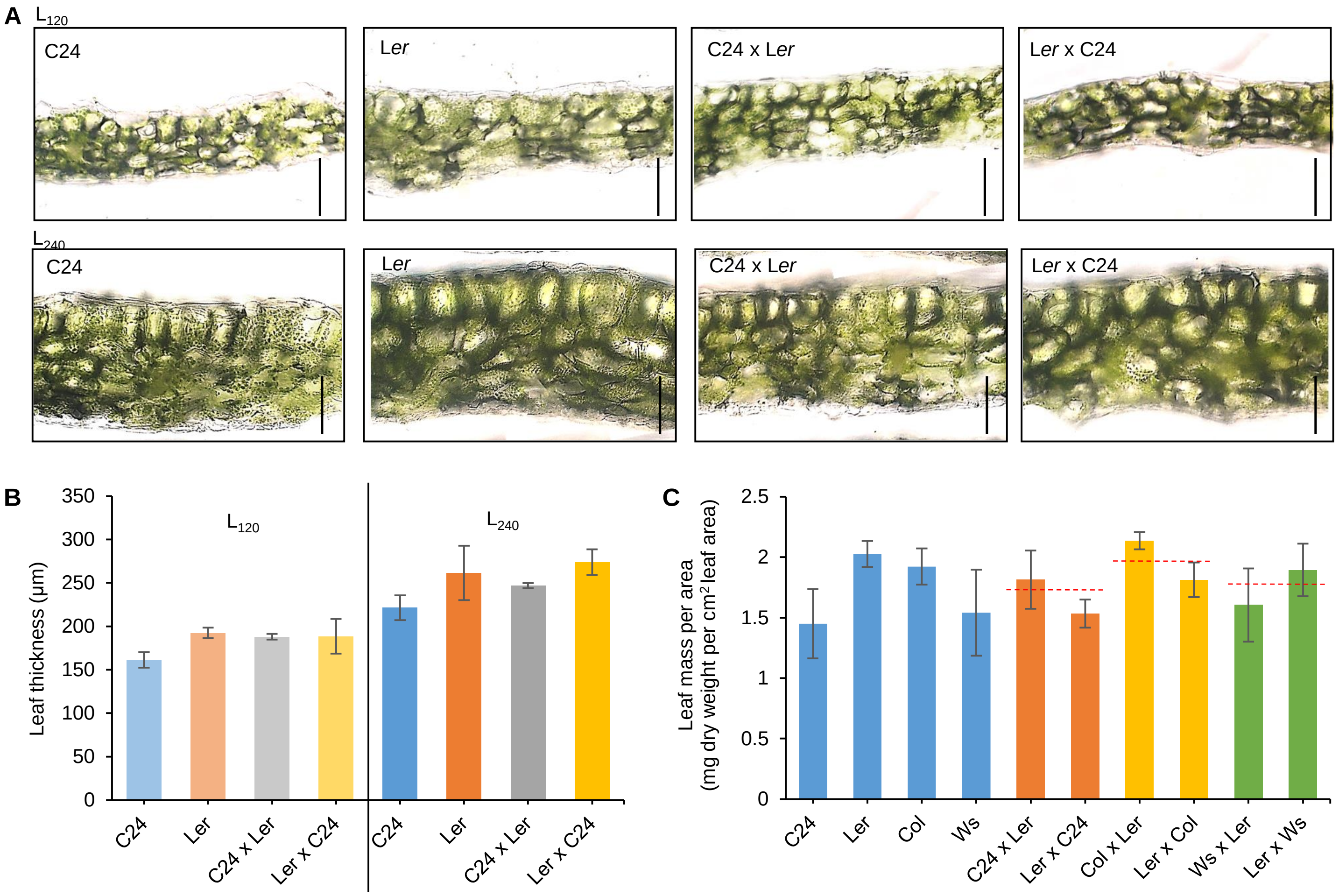

Supplementary Figure 2. Comparisons of leaf morphology between hybrids and parents. (A) The leaf cross-sections of the 4 week-old C24/*Ler* hybrid and parents grown under 16hr photoperiod under 120 µmol m^{-2} s^{-1} (L_{120}) or 240 µmol m^{-2} s^{-1} (L_{240}; representative of n=3). The largest leaves (leaves 5 and 6) were examined under a microscope (20X objective). The leaf cross-sections were presented with the central midvein aligning to the left and the adaxial epidermis to the top. Scale bar represents 200 µm. (B) Comparisons of leaf thickness of the C24/Ler hybrid and parents. Leaf thickness was determined by measuring the distance between the upper and lower epidermis using ImageJ. Data presented were the average and SE of n=3. (C) Comparison of leaf mass per area between hybrids and parents grown under 16hr photoperiod under 120 µmol m^{-2} s^{-1} . Data shown were calculated using data sets measured from leaves 5 or 6 of 4 week-old, soil-grown plants. Each leaf sampled was oven-dried and determined for dry weight. Leaf area was determined from the leaf image taken upon harvesting using ImageJ. Each data set represents the average and standard error of n=5 plants. No significant differences were found between hybrids and the average of the parents (ANOVA, $p > 0.05$).

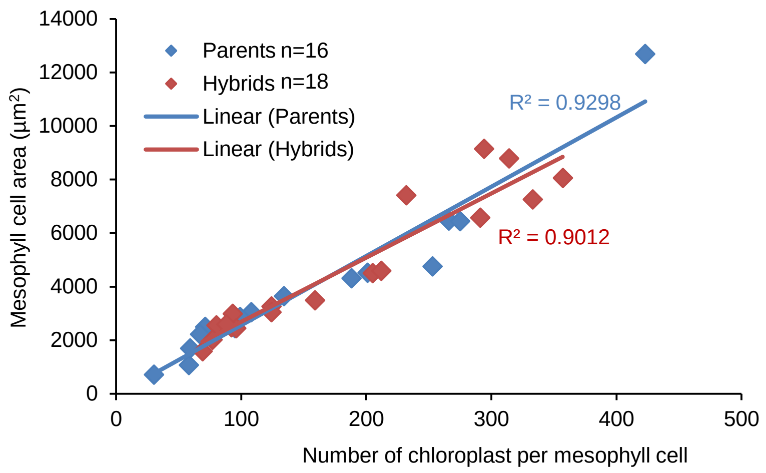

Supplementary Figure 3. Comparisons of chloroplast content between the C24/Ler hybrids and parents. The coefficient of definition of the regression (black line). The mesophyll cells from the cotyledons and true leaf 1/2 of C24/Ler hybrid seedlings at 10 DAS and 19 DAS, respectively, were examined individually using Nomaski light microscopy and the number of chloroplasts was counted. The cell area was determined using ImageJ. R^2: The coefficient of definition of the regression (black line). Data presented are the average and SE from number of replicates as indicated.

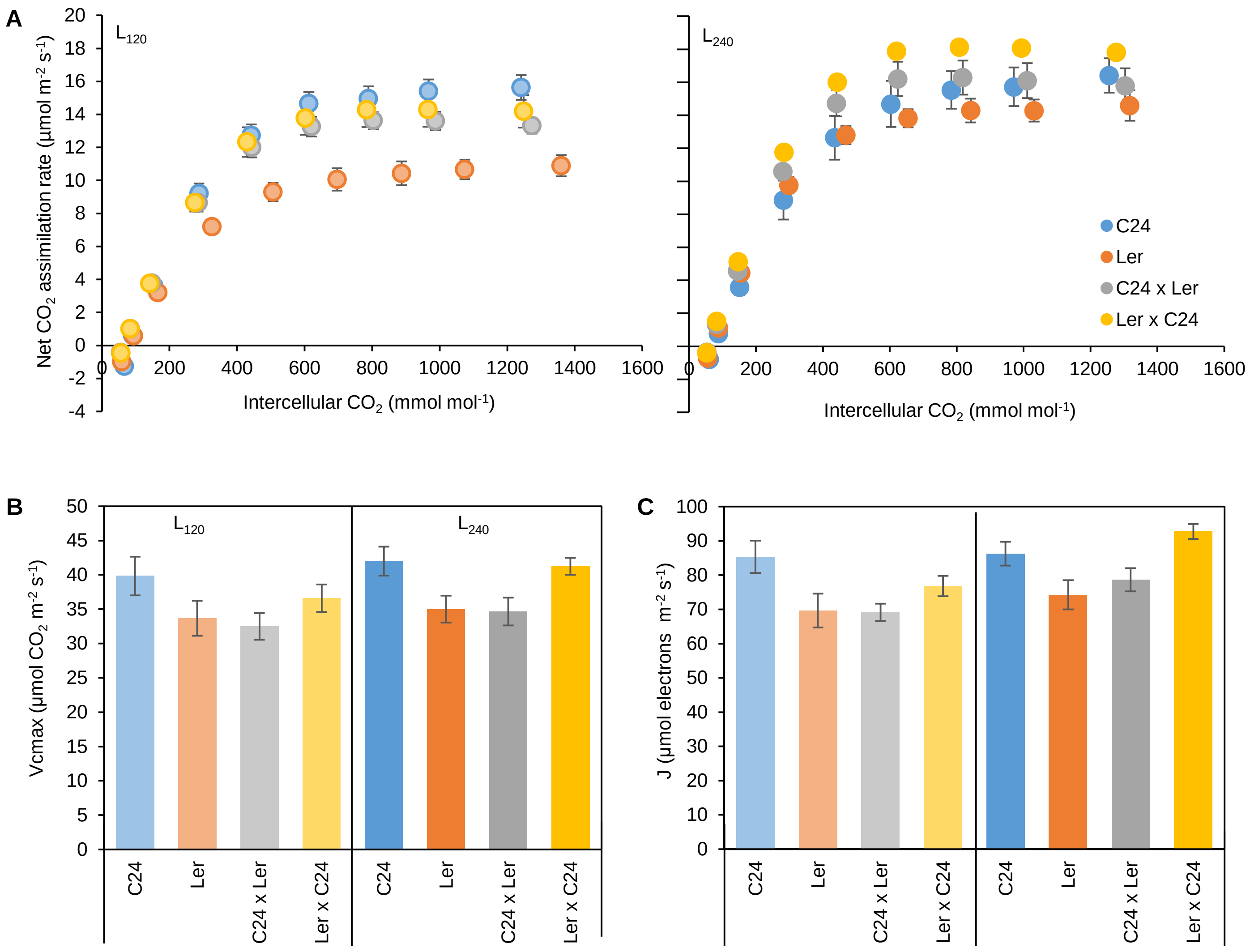

Supplementary Figure 4. Photosynthetic parameters of 240 µmol m^{-2} s^{-1}-grown C24/ *Ler* hybrids and parents. (A) A/Ci curves of the 3 week-old C24/Ler seedlings grown under 120 µmol m^{-2} s^{-1} (L_{120}) or 240 µmol m^{-2} s^{-1} (L_{240}) under a light-dark cycle of 16hr/ 8hr at 21 °C. Analyses of CO_2 assimilation in an ascending atmospheric CO_2 partial pressure were carried out under a saturating light (1000 µmol m^{-2} s^{-1}) using a gas exchange analyser (LI-6400XT, LICOR). Data presented were the average and SE of n ≥ 3 replicates. A: net CO_2 assimilation; Ci: Intercellular CO_2 partial pressure. (B)(C) Estimated photosynthetic parameters derived from the A/Ci curves by fitting to C3 photosynthesis model. Data represents the average and SE of maximum rate of Rubisco carboxylation (V_{cmax}) and estimated electron transport rate (J) determined from n ≥ 3.

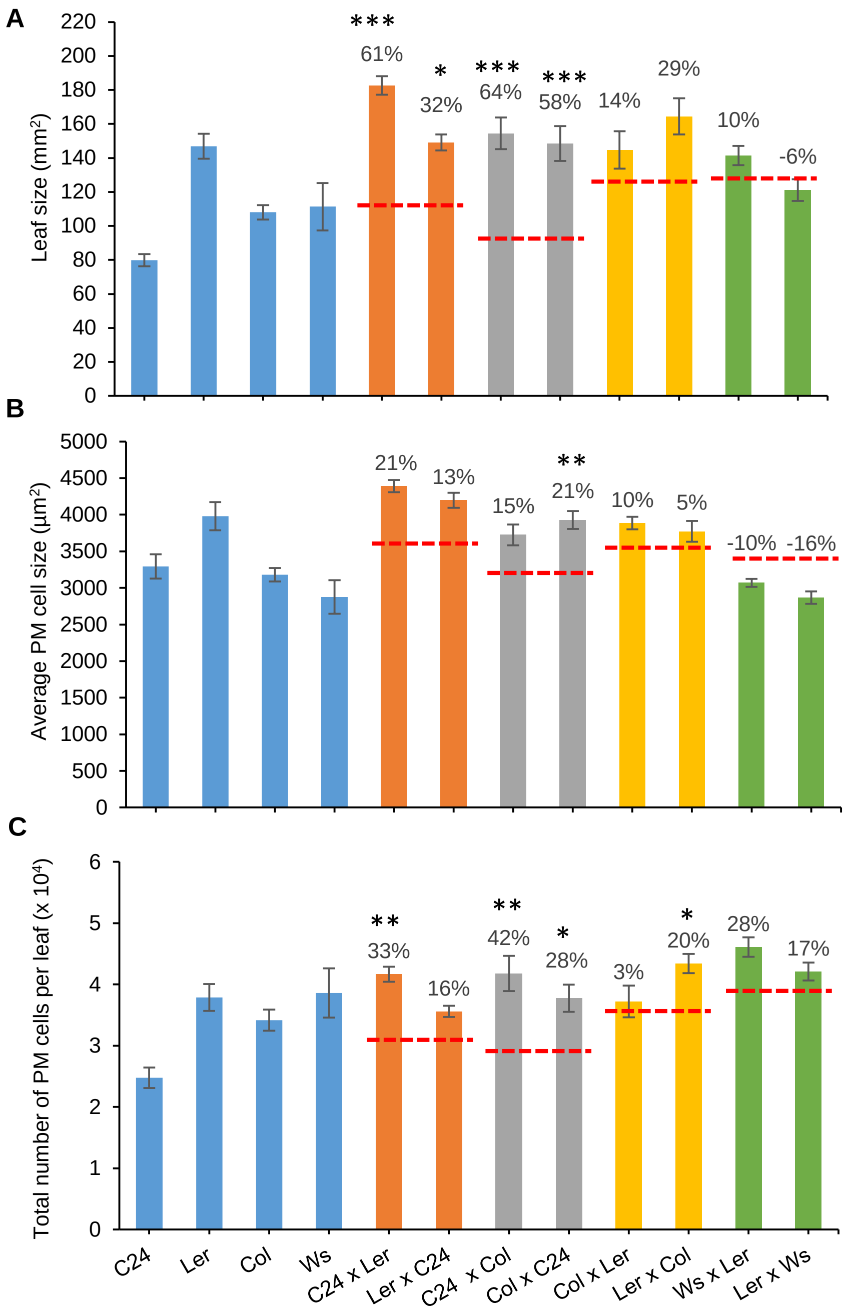

Supplementary Figure 5. Cellular basis of leaf size differences between hybrids and parents. The fully expanded leaves 3/4 of the 28 DAS seedlings were used. For each leaf analysed, microscopic images of palisade mesophyll layer were taken at six positions and the average cell number per image was calculated. The average area of individual palisade mesophyll cell was determined by dividing the known leaf area in a microscopic image by the average cell number per image, and the estimated total number of palisade mesophyll cells per leaf was calculated by dividing the total leaf area by the average palisade mesophyll cell area. Data presented were the average and SE of n=7-12 leaves from 3-6 plants. Values above columns represent percentage increase from the average of the parents (red dashed lines). Asterisks indicate significant increase compared with the average levels of the parents (ANOVA; *: $p < 0.05$, **: $p < 0.01$, ***: $p < 0.001$). PM: palisade mesophyll.

Supplementary Table 1. Chlorophyll content of the 4 week-old C24/L*er* hybrid seedlings grown under a 16hr-photoperiod. The L_{240}-grown plants were grown in 120 µmol m^{-2} s^{-1} for 10 days before transferring into 240 µmol m^{-2} s^{-1}. Pools of rosettes (three repeats of n=3; standard error) were analysed for chlorophyll content per unit fresh weight. Statistical comparisons were carried out between hybrids and parents; and amongst plants growing under different light conditions (ANOVA). No significant differences were found ($p > 0.05$). a/ b ratio: chlorophyll a to b ratio.

Light condition	Line	Chl a (µg/ mg FW)	Chl b (µg/ mg FW)	Chl a/ b ratio
L_{120}	C24	2.36 ± 0.09	1.14 ± 0.05	2.1 ± 0.02
	Ler	2.44 ± 0.05	1.19 ± 0.05	2.1 ± 0.05
	C24 x Ler	2.46 ± 0.09	1.20 ± 0.04	2.1 ± 0.02
	Ler x C24	2.49 ± 0.05	1.21 ± 0.04	2.1 ± 0.013
L_{240}	C24	2.19 ± 0.04	0.99 ± 0.02	2.2 ± 0.02
	Ler	2.19 ± 0.09	1.06 ± 0.04	2.1 ± 0.13
	C24 x Ler	2.29 ± 0.05	1.15 ± 0.11	2.0 ± 0.18
	Ler x C24	2.24 ± 0.06	1.01 ± 0.02	2.2 ± 0.01

Supplementary Table 2. Leaf number and flowering time of C24/L*er* hybrids and parents. Data is the average and SE of n = 5-12 seedlings that were randomly selected from a population of n=30 plants grown under 16hr photoperiod in 120 µmol m^{-2} s^{-1} (L_{120}) or 240 µmol m^{-2} s^{-1} (L_{240}) at 21°C. Flowering time was determined by the bolting of shoot (about 1 cm). ANOVA was used to compare between each reciprocal hybrid to one of the parents. Significant differences to at least one of the parents (15 DAS L*er*; 18 DAS C24 and 30 DAS L*er*) are indicated by black asterisks and red asterisks indicate the hybrid is significantly different from both parents. DAS: days after sowing.

Line	Number of leaves				Days to flower	
	15 DAS	18 DAS	21 DAS	30 DAS	16h/ L_{120}	16h/ L_{240}
C24	6 ± 0.2	8 ± 0.4	11 ± 0.2	14 ± 1.5	31 ± 1.4	36 ± 0.7
Ler	6 ± 0.2	9 ± 0.4	8 ± 0.4	8 ± 0.7	24 ± 1.1	25 ± 0.5
C24 x Ler	7 ± 0.3 *	9 ± 0.3	13 ± 0.5 *	19 ± 1.7 *	38 ± 0.5 *	42 ± 0.6 *
Ler x C24	7 ± 0.3 *	10 ± 0.5 *	12 ± 0.4 *	21 ± 0.7 *	39 ± 0.7 *	41 ± 1.8 *

Supplementary Table 3. Measurements of palisade mesophyll cells of leaves 3/4 of 19 DAS and 28 DAS and leaves 5/6 of 19 DAS C24/*Ler* hybrid plants compared with the parents. Number of cells was determined under a microscope (20x objective, field area: 660 µm x 528 µm). Data were the average and SE from n= 6 leaves collected from three plants grown in a light/dark cycle of 16hr/ 8hr under 120 µE at 21°C. Asterisks indicate significant difference relative to the best parent (ANOVA, $p < 0.05$).

**Leaf area (cm²)**	**28 DAS Leaf 3/4**	**19 DAS Leaf 3/4**	**19 DAS Leaf 5/6**
C24	0.79 ± 0.04	0.33 ± 0.03	0.21 ± 0.03
Ler	1.47 ± 0.07	0.43 ± 0.03	0.22 ± 0.03
C24 x *Ler*	1.83 ± 0.05 *	0.55 ± 0.03 *	0.37 ± 0.07 *
Ler x C24	1.49 ± 0.05	0.45 ± 0.01	0.24 ± 0.03

**Number of cells per fixed area**	**28 DAS Leaf 3/4**	**19 DAS Leaf 3/4**	**19 DAS Leaf 5/6**
C24	118 ± 5	261 ± 15	214 ± 50
Ler	83 ± 3	279 ± 31	194 ± 43
C24 x *Ler*	87 ± 2	221 ± 28	175 ± 33
Ler x C24	91 ± 2	243 ± 5	201 ± 48

**Average cell size (µm²)**	**28 DAS Leaf 3/4**	**19 DAS Leaf 3/4**	**19 DAS Leaf 5/6**
C24	3294 ± 167	1354 ± 72	506 ± 92
Ler	3981 ± 191	1311 ± 139	559 ± 102
C24 x *Ler*	4391 ± 84	1723 ± 250	811 ± 203
Ler x C24	4198 ± 104	1436 ± 29	567 ± 114
